# Data integration aids understanding of butterfly–host plant networks

**DOI:** 10.1038/srep43368

**Published:** 2017-03-06

**Authors:** Ai Muto-Fujita, Kazuhiro Takemoto, Shigehiko Kanaya, Takeru Nakazato, Toshiaki Tokimatsu, Natsushi Matsumoto, Mayo Kono, Yuko Chubachi, Katsuhisa Ozaki, Masaaki Kotera

**Affiliations:** 1 Graduate School of Biological Sciences, Nara Institute of Science and Technology (NAIST), 8916-5 Takayama, Ikoma, Nara 630-0192, Japan; 2 Department of Bioscience and Bioinformatics, Kyushu Institute of Technology, Kawazu 680-4, Iizuka, Fukuoka 820-8502, Japan; 3 Graduate School of Information Sciences, Nara Institute of Science and Technology (NAIST), 8916-5 Takayama, Ikoma, Nara 630-0192, Japan; 4 Database Center for Life Science (DBCLS), Research Organization of Information and Systems, Yata 1111, Mishima, Shizuoka 411-8540, Japan; 5 DDBJ Center, National Institute of Genetics, Research Organization of Information and Systems, Yata 1111, Mishima, Shizuoka 411-8540, Japan; 6 Neko-System Inc., 8-31, Konyamachi, Takatsuki, Osaka 569-0804, Japan; 7 School of Life Science and Technology, Tokyo Institute of Technology, 2-12-1 Ookayama, Meguro-ku, Tokyo 152-8550, Japan; 8 JT Biohistory Research Hall, 1-1 Murasaki-cho, Takatsuki, Osaka 569-1125, Japan

## Abstract

Although host-plant selection is a central topic in ecology, its general underpinnings are poorly understood. Here, we performed a case study focusing on the publicly available data on Japanese butterflies. A combined statistical analysis of plant–herbivore relationships and taxonomy revealed that some butterfly subfamilies in different families feed on the same plant families, and the occurrence of this phenomenon more than just by chance, thus indicating the independent acquisition of adaptive phenotypes to the same hosts. We consequently integrated plant–herbivore and plant–compound relationship data and conducted a statistical analysis to identify compounds unique to host plants of specific butterfly families. Some of the identified plant compounds are known to attract certain butterfly groups while repelling others. The additional incorporation of insect–compound relationship data revealed potential metabolic processes that are related to host plant selection. Our results demonstrate that data integration enables the computational detection of compounds putatively involved in particular interspecies interactions and that further data enrichment and integration of genomic and transcriptomic data facilitates the unveiling of the molecular mechanisms involved in host plant selection.

Herbivorous insects and their host plants have been engaged in a chemical arms race for more than 420 million years^1^. Whilst host plants have developed various chemicals for defence against herbivorous insect damage, herbivorous insects have evolved various countermeasures^2^,^3^. Some insects even use plant chemicals for their own benefit^4^. Most chemical ecology studies have focused on a small number of specific species rather than systematically exploring a wide range of species.

Many herbivorous butterflies feed on specific host plants, thus forming plant–herbivore networks. In recent years, large-scale data on ecological networks, including plant–herbivore networks, have become available with the development of new observation techniques and with improvements in databases and other infrastructure. Network analysis techniques resulting from the development of network science^5^ have been used in ecology to actively investigate ecological networks, with respect to both basic scientific research (e.g., structure–stability relationships) and applied ecology (e.g., biodiversity maintenance and environmental assessment)^6^,^7^,^8^.

Taxonomic families of herbivorous butterflies generally correspond to those of host plants, thus reflecting the close co-evolutionary relationship between butterflies and plants. A correlation has been observed between changes in host plants and species diversification in the butterfly superfamily Papilionidea^9^. Numerous observations of host–plant selection have established that this selection is controlled by chemical constraints, i.e., several insect species recognise plants by detecting their chemical components^10^,^11^,^12^,^13^,^14^,^15^,^16^. Nevertheless, the molecular mechanisms that determine host–plant shifts are poorly understood^16^. A general understanding of the molecular factors involved in host–plant selection requires detailed chemical and genomic studies on a wide range of insects and plants.

To address this problem, we integrated biological data to gain a comprehensive picture of the contribution of plant chemical compounds to host plant selection and to identify candidate plant compounds involved in specific interactions between butterfly and plant families. This required us to first comprehensively collect data on plant–herbivore relationships involving Japanese butterflies, which were not previously available in the HOSTS lepidopteran host plant database [ http://www.nhm.ac.uk/our-science/data/hostplants/].

The Japanese Islands lie at the convergence of three terrestrial ecozones, namely, the Palearctic (including Europe, Asia north of the Himalayan foothills, northern Africa, and the northern and central Arabian Peninsula), the Oriental (extending across most of South and Southeast Asia and into southern East Asia), and Oceania. Although the flora and fauna of the Japanese Islands are influenced by all three ecozones, they retain their unique status in these islands, which extend from the subarctic to subtropical zones and include many highlands. These geographic and ecological features, as well as an abundance of accumulated knowledge from observations of area-specific populations of butterflies, are attractive targets for research on species diversification. We have generated a complete plant–herbivore network for the Japanese Islands. This network, designated the InsectInDB database (http://insect-plant.org/), comprises 545 insects, 1,922 plants and 3,435 insect–plant relationships.

In this study, we used network analysis combined with taxonomic information to clearly demonstrate that butterfly subfamilies in different families feed on the same plant groups, thereby indicating the independent acquisition of the same adaptive phenotypes (i.e., to the same hosts). The incorporation of chemical compound data allowed us to consider the contributions of plant compounds to butterfly host–plant selection by comparing two hypotheses: a “feed-on-family” hypothesis in which butterflies preferentially feed on host plants in the same family, and a “feed-on-compound” hypothesis in which butterflies select plants that produce similar compounds. Statistical analysis enabled the identification of compounds specific to host plants of particular butterfly families. We determined that some of these compounds are known to attract limited groups of butterflies and repel others. An integration of the insect–compound relationship data revealed potential metabolic processes related to host-plant selection. Our results successfully demonstrated the value of data integration for the computational detection of candidate host plant selection compounds. Finally, the further integration of genomic and transcriptomic data allowed us to unveil the putative molecular mechanism of host plant selection.

## Results

### Study strategy overview

**Figure 1:**
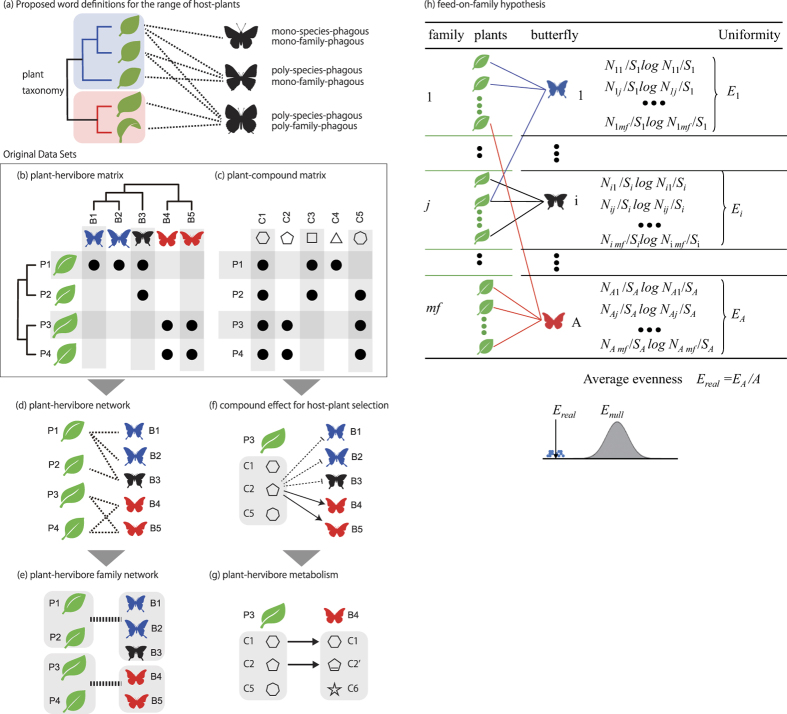
Overview of the strategy used in this study. (**a**) Proposed word definitions to describe the range of host-plant preferences. (**b**) Plant–herbivore matrix. Rows and columns represent plants and herbivorous butterflies, respectively. A dot at an intersection indicates that the butterfly feeds on the corresponding plant. Butterfly taxonomy is indicated by the dendrogram. (**c**) Plant–compound matrix. Rows and columns represent plants and compounds, respectively. Dots are used to indicate that a plant possesses a compound. Plant taxonomy is indicated by the dendrogram. (**d**) Plant–herbivore network consisting of butterfly species with their host plant species as the nodes. (**e**) Plant–herbivore family network consisting of butterfly families with their host plant families as the nodes. (**f**) Compound effect on host plant selection. Arrows and dotted lines indicate that a plant compound attracts or repels herbivorous butterflies, respectively. (**g**) Plant–herbivore metabolism. Some compounds (such as C1) found in herbivorous butterflies are believed to be directly obtained from the host plant, whereas other compounds (such as C2′) are derived from plant compounds via enzymatic reactions or from other sources (such as C6). (**h**) Feed-on-family hypothesis.

Before describing the approach used in this study, we first will define the terms used in this paper to describe different plant–insect relationships. Several sets of words, including generalist/specialist and monophagous/oligophagous/polyphagous, have been used to represent the range of insect host preferences^17^. When discussing plant–herbivore relationships, a specialist is an herbivore that has evolved a specific mechanism to feed on a particular host plant, whereas a generalist is one that has not. Monophagous, oligophagous and polyphagous refer to the number of host plants fed on, i.e., only one, a few, or many host plants, respectively. Alternatively, oligophagous is defined as the use of multiple host plant species from the same family^18^. Generalist/specialist can also refer to the breadth of plant selection by pollinator insects^19^. In many cases, however, these words are ambiguous. For example, “specialist” and “monophagous” typically indicate that an herbivore feeds on a single host plant species, but they are sometimes applied to an herbivore feeding on an entire family. To avoid confusion, we define the number of host plant species and host plant families as “mono-species-phagous/oligo-species-phagous/poly-species-phagous” and “mono-family-phagous/oligo-family-phagous/poly-family-phagous”, respectively (Fig. 1a). Similar to oligophagous/polyphagous, the distinction between “oligo-” (several) and “poly-” (many) is somewhat subjective. In this paper, we use these words to avoid ambiguity when discussing plant–herbivore relationships at the species and family levels.

To achieve a general understanding of host plant selection by Japanese butterflies, we considered plant–herbivore relationships (Fig. 1b) and the presence of compounds in various plant species (Fig. 1c) as follows. First, plant–herbivore relationships between butterflies and their host plants were represented as a bipartite network (Fig. 1d) in which a node represents a butterfly or plant species and an edge indicates that the butterfly feeds on the host plant. Second, the network was divided into sub-networks according to taxonomic family (Fig. 1e). The combined use of plant–herbivore relationship (Fig. 1b) and plant–compound relationship (Fig. 1c) data allowed us to identify compounds common to a group of host plants that are specific to a group of butterflies (in order to distinguish from just “common” compounds, we refer to this type of compounds as “common specific” compounds in this paper). For example, suppose that butterflies B4 and B5 feed specifically on plants P3 and P4 (Fig. 1b). Among the compounds shown in Fig. 1c, C1 is “*common*” to all plants, which indicates that C1 may be important for plant survival in general but may not be involved in interactions with specific butterflies. Compound C2 is *common specific* to plants P3 and P4 but not P1 and P2, indicating the possibility that C2 functions to attract butterflies B4 and B5 and repel B1, B2 and B3 (Fig. 1f). When we examined the contributions of these *common specific* compounds to host plant selection, we found that some were, in fact, important in the plant–herbivore relationship. Finally, a comparison of compounds identified as participants in plant–herbivore relationships unveiled insect metabolic processes that may be involved in the interaction (Fig. 1g).

### General architecture of the Japanese butterfly plant–herbivore network

**Figure 2:**
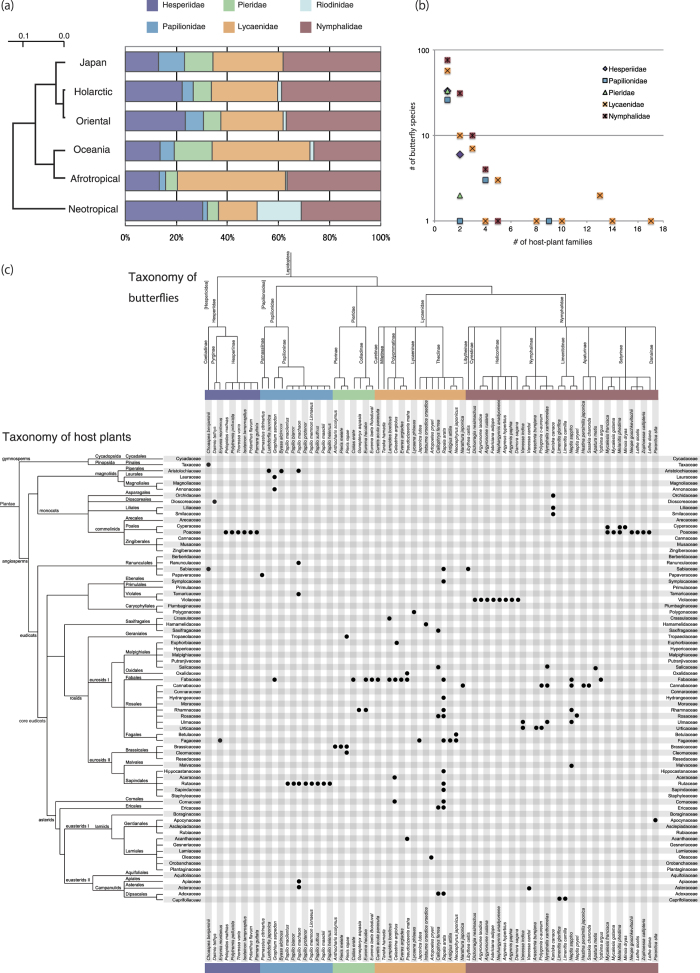
(**a**) Distribution of butterfly families across the Japanese Islands and other global ecozones. (**b**) Distribution of host plants among butterfly families. Horizontal and vertical axes represent the number of host plant families per butterfly species and the number of butterfly species, respectively. (**c**) Plant–herbivore matrix of some Japanese butterfly species. Mono-family-phagous butterflies are *Daimio tethys, Erynnis montanus, Pelopidas mathias, Polytremis pellucida, Thoressa varia, Isoteinon lamprospilus, Potanthus flavum, Parnara guttata, Parnassius citrinarius, Luehdorfia japonica, Byasa alcinous, Papilio macilentus, Papilio bianor, Papilio protenor, Papilio memnon Linnaeus, Papilio xuthus, Papilio maackii, Papilio helenus, Anthocharis scolymus, Pieris melete, Colias erate, Gonepteryx aspasia, Eurema laeta Boisduval, Curetis acuta paracuta, Everes argiades, Lycaena phlaeas, Japonica lutea, Iratsume orsedice orsedice, Artopoetes pryeri, Antigius attilia, Narathura japonica, Libythea celtis, Dichorragia nesimachus, Argyronome laodice, Argyronome ruslana, Fabriciana adippe, Nephargynnis anadyomene, Argyreus hyperbius, Argynnis paphia, Damora sagana, Vanessa cardui, Araschnia burejana, Limenitis glorifica, Limenitis camilla, Neptis pryeri, Hestina persimilis japonica, Sasakia charonda, Apatura metis, Ypthima argus, Mycalesis gotama, Minois dryas, Neope goschkevitschii, Lethe sicelis, Zophoessa callipteris, Lethe diana, Parantica sita*. Oligo-family-phagous butterflies are *Choaspes benjaminii, Graphium sarpedon, Pieris rapae, Eurema hecabe, Lampides boeticus, Pseudozizeeria maha, Neozephyrus japonicus, Vanessa indica, Polygonia c-aureum, Kaniska canace, Mycalesis francisca, Melanitis phedima*. Poly-family-phagous butterflies are *Papilio machaon, Celastrina argiolus, Callophrys ferrea, Rapala arata, Nymphalis xanthomelas, Neptis sappho. Taraka hamada* is not herbivore but carnivore.

Several seminal studies have revealed that taxonomically related host plants are used by particular butterfly families^20^,^21^. In the present study, we adopted the most recently reported phylogenies of angiosperm plants (APGIII)^22^ and butterflies^23^ to examine the relationships between butterfly–host plant associations in the Japanese Islands. To analyse the network architecture as a whole, we conducted a comprehensive literature survey in which 3,435 plant–herbivore relationships encompassing 1,922 plant species and all 545 butterfly species living in Japan were examined. All members of the superfamily Rhopalocera except for Riodinidae are found in the Japanese Islands; these butterflies are classified into the families Hesperiidae, Papilionidae, Pieridae, Lycaenidae and Nymphalidae. Figure 2a shows the distribution of butterfly families in the Japanese Islands and global ecozones. We conducted a similarity analysis based on Pearson’s correlation coefficients (*r*) of the relative proportions of butterfly families in different ecozones, which indicated that the butterfly composition of the Japanese Islands is similar to those of the Holarctic (*r* = 0.92) and Oriental (*r* = 0.91) ecozones. This result may imply that butterfly distributions in the Japanese Islands are more influenced by distributions in the Holarctic and Oriental ecozones than by those in Oceania or other ecozones, however, it still lacks solid evidence and analysis. The number of butterfly species in Japan (302) is much smaller than the numbers in other ecozones (2,224, 2,411, 1,274, 3,964 and 7,784 in Holarctic, Oriental, Oceania, Afrotropical and Neotropical ecozones, respectively). Another factor that may need to be considered is that Papilionidae butterflies are especially popular in Japan and have been extensively catalogued.

Figure 2b, which shows the relationship between the number of host-plant families and butterfly species, clearly demonstrates that host plant preferences differ among butterfly families. For example, Hesperiidae and Lycaenidae both feed on a wide range of host plant species; however, the butterflies in the first family feed on members of one or two specific host plant families, whereas butterflies in the latter family feed on a wider variety of host plant families. To distinguish between these behaviours, we therefore prefer to use mono-species-phagous/oligo-species-phagous/poly-species-phagous and mono-family-phagous/oligo-family-phagous/poly-family-phagous, as proposed above, rather than the existing terms, specialist/generalist and monophagous/oligophagous/polyphagous. The matrix representation in Fig. 2c clearly reveals some characteristics of the host plant selectivity of Japanese butterflies. For example, the genus *Papilio* in Papilionidae and the subfamilies Pierinae in Pieridae and Heliconiinae in Nymphalidae are generally mono-family-phagous and have specific relationships with host plants in Rutaceae, Brassicaceae and Violaceae, respectively. In contrast, some butterfly subfamilies in different families exhibit shared host plant selectivity: for example, the subfamily Coliadinae in Pieridae and the subfamily Polyommatinae in Lycaenidae both feed on plants in Fabaceae, whereas the subfamily Hesperiinae in Hesperiidae and the subfamily Satyrinae in Nymphalidae both interact with host plants in Poaceae. Poly-family-phagous butterflies can also be identified from the matrix. For example, butterflies in the subfamily Theclinae in Lycaenidae generally feed on more than three host-plant families.

**Figure 3:**
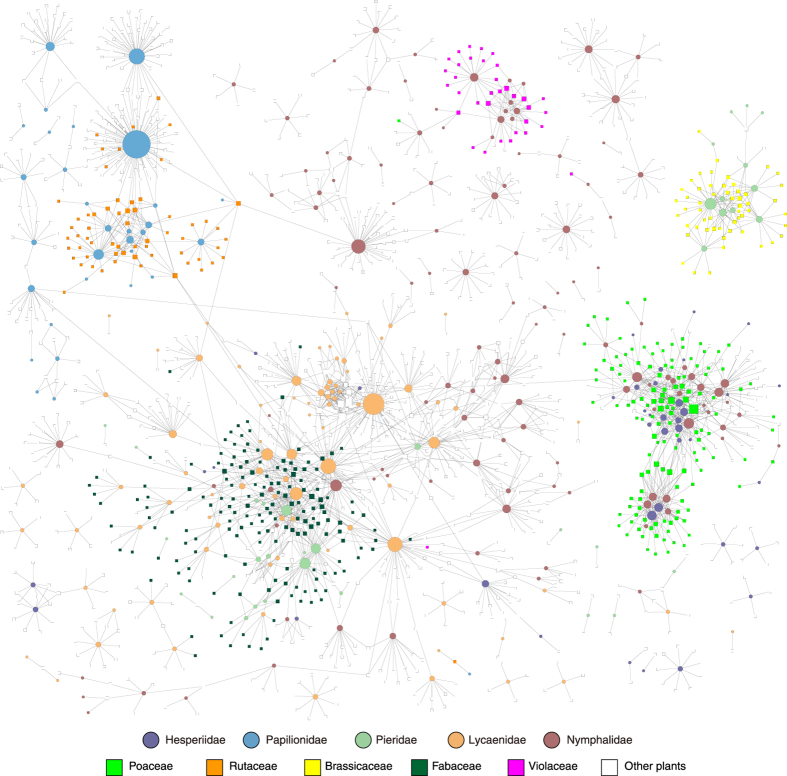
Plant–herbivore network of all Japanese butterfly species. Circles and rectangles represent butterfly and plant species, respectively, with the edges between them representing their relationship. Nodes (circles and rectangles) are coloured to indicate families of butterflies and plants, respectively. Node sizes are proportional to degree (i.e., number of edges per node).

An examination of the entire network of plant–herbivore relationships (Fig. 3) also reveals that butterfly species in the same family tend to share the same host-plant species and families. The Papilionidae, Nymphalidae and Pieridae families have their own predominant sub-networks (upper left, upper middle and upper right in Fig. 3, respectively). Rutaceae and Brassicaceae are examples of plant families predominantly fed on by single butterfly families (Papilionidae and Pieridae, respectively). In contrast, two or more butterfly families (Hesperiidae, Lycaenidae and Nymphalidae) share for the plants of the families Poaceae and Fabaceae.

**Figure 4:**
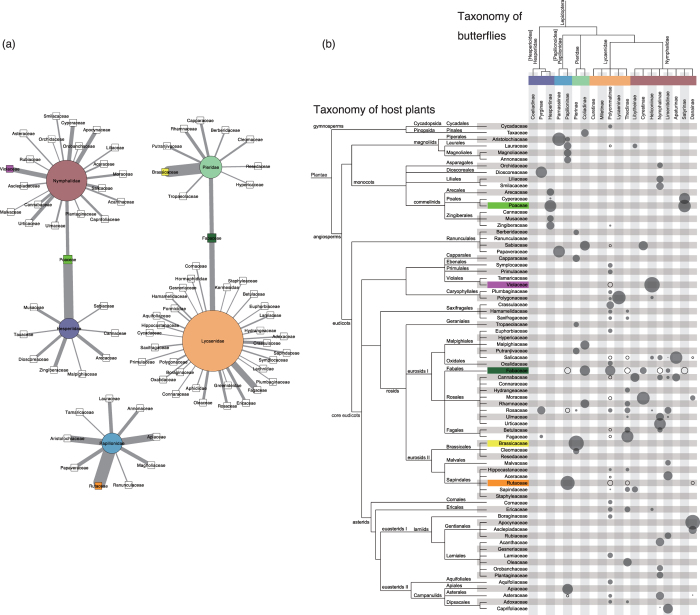
(**a**) Family-level plant–herbivore network. Circles and rectangles represent butterfly and plant families, respectively, with the edges between them representing their relationships. Node sizes are proportional to degree (i.e., number of edges per node). Edge widths represent the *Z*-scores. Node colours (circles and triangles) indicate that the butterfly and plant families are the same as in Fig. 3. (**b**) Plant–herbivore matrix of plant families and butterfly subfamilies. Closed and open circles indicate plant–herbivore relationships with positive and negative interaction frequency *Z*-scores, respectively. Areas of circles are proportional to *Z*-score absolute values.

To further analyse plant–herbivore relationships, we simplified the network to describe significant interactions between plant and butterfly families (Fig. 4a) with significance evaluated based on real vs. randomised network *Z*-scores (see Methods). Positive *Z*-scores indicated that plants in a particular family are significantly more likely to be eaten by insects from a certain family than by those in the randomised networks. As shown in Fig. 4a, the five butterfly families clearly form selective plant–herbivore relationships. For example, Papilionidae butterflies interact specifically with the families Rutaceae, Apiaceae, Aristolochiaceae and Lauraceae, thus forming an isolated network. The remaining four butterfly families also have their own specific host plant families, with two exceptions: Coliadinae subfamily in Pieridae and Polyommatinae subfamily in Lycaenidae butterflies share Fabaceae plants, and Hesperiinae subfamily in Hesperiidae and Satyrinae subfamily in Nymphalidae butterflies share plants in Poaceae. These butterfly subfamilies are in different families. Nonetheless, the families have independently acquired the ability to use the same groups of plants as hosts.

Figure 4b shows significant plant–herbivore relationships between plant families and butterfly subfamilies. For example, the two subfamilies of Papilionidae (Parnassiinae and Papilioninae) have significantly different host plant families, and the same is true for the two Pieridae families (Pierinae and Coliadinae). Among the butterfly subfamilies in Lycaenidae, Curetinae and Miletinae have no specific host plant families, Polyommatinae and Theclinae are poly-family-phagous, and Lycaeninae is mono-family-phagous. The subfamily Satyrinae in Nymphalidae and the subfamily Hesperiinae in Hesperiidae might be regarded as generalist taxa because they feed on two or more host plant families. Because their host-plants are mostly restricted to a single order, we instead consider these latter butterflies to be mono-order-phagous.

With respect to butterfly families (Fig. 4a), the plant families Poaceae and Fabaceae are significantly shared by two butterfly families. At the butterfly subfamily level (Fig. 4b), plants in Fabaceae are consumed by the subfamily Coliadinae in Pieridae and by the subfamily Polyommatinae in Lycaenidae. Members of Poaceae serve as host plants for both the subfamily Satyrinae in Nymphalidae and the subfamily Hesperiinae in Hesperiidae. We found that these two plant families differ with respect to the types of butterflies that consume them. In particular, Poaceae is significantly eaten by subfamilies that are mono-order-phagous (Hesperiinae and Satyrinae), whilst Fabaceae is eaten at a significant level by poly-family-phagous subfamilies (Coliadinae, Polyommatinae, Cyrestinae and Limenitidinae).

A negative *Z*-score indicates that plants belonging to a particular family are significantly less subject to predation by a given insect family compared with those in randomised networks. Thus far, we have shown that Poaceae and Fabaceae are eaten by a wide range of butterflies (Fig. 4a). However, having analyzed the relationships with butterfly subfamilies, we also found that Poaceae and Fabaceae are consumed differently (Fig. 4b). Poaceae members have no significant relationships exhibiting negative *Z*-scores, indicating that there are no butterfly subfamilies that significantly avoid Poaceae plants. On the other hand, Fabaceae members have some relationships exhibiting negative *Z*-scores, i.e., significantly less numbers of host-plant relationships with Papilioninae, Theclinae, Nymphalinae and Satyrinae. To explain this difference, we hypothesised the presence/absence of some factors that work as repellent for some butterflies but as attractant for some other butterflies. In other words, Fabaceae plants are eaten by a wide range of butterflies because they possess some factors that exert both significantly positive (i.e., attractive) and negative (i.e., repellent) effects, whereas Poaceae plants are devoured by a wide range of butterflies because they have attraction factors but not repellent factors.

### Influence of plants on host-plant selection

In the previous section, we demonstrated that host-plant selection is highly associated with butterfly taxonomic classifications and implied the existence of factors influencing host-plant selection. To provide more conclusive evidence for this idea, we explored two possible hypotheses: the “feed-on-family” hypothesis or the “feed-on-compound” hypothesis (Fig. 1h). To test the likelihood of the feed-on-family hypothesis, we calculated *Z*-score statistics based on family-based evenness for butterfly category (family or subfamily) *i, E*^real^(*i*), and the evenness of randomised networks, *E*^rand^(*i*) (see Methods). We found that the average *E*^real^(*i*) (0.03) was significantly lower than the average *E*^rand^(*i*) (0.16) (*Z* = –61.9; *p* < 2.2 × 10^–16^ using the *Z*-test).

We likewise examined the possibility of the feed-on-compound hypothesis. To determine whether a set of host plants of a butterfly species tended to possess a higher number of common specific compounds compared with those from randomly selected sets, we applied the food-pairing hypothesis^24^ to our datasets. For the statistical test, we used the average number of compounds shared among host plants by butterfly species *k, N*_*s*_(*k*)^24^. From the original plant–herbivore network, we extracted all plant–herbivore relationships in which at least one compound was present in the host plant according to the species-metabolite relationship database KNApSAcK [25]. This extracted network, which consists of 216 butterfly and 405 plant species, is subsequently referred to as the real-world network. A comparison of real-world and randomised network *N*_*s*_(*k*) values (*N*_*s*_^real^(*k*) and *N*_*s*_^rand^(*k*), respectively) revealed that the average *N*_*s*_^real^ (*k*) (0.37) was significantly larger than the average *N*_*s*_^rand^ (*k*) (0.09) (*Z* = 8.9; *p* < 2.2 × 10^–16^ using the *Z*-test).

**Figure 5:**
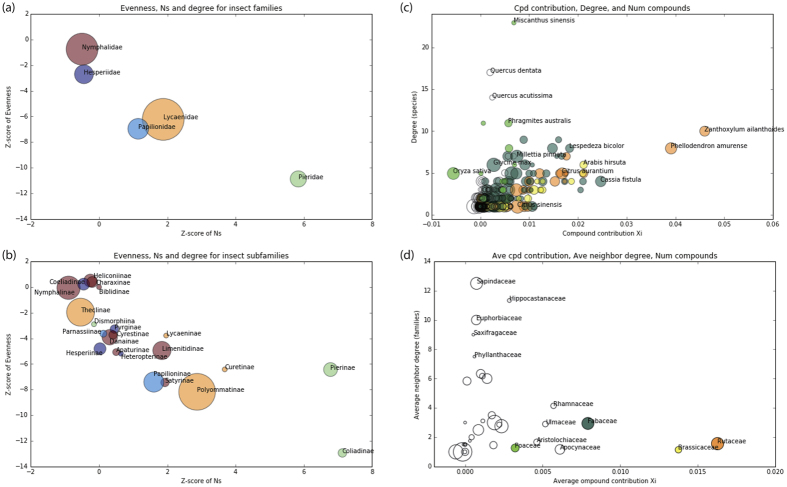
(**a**) Relationships among *Z*-scores of average plant family-based evenness for butterfly families and Z-scores of the average number of common specific compounds in host plants. Circles represent butterfly families. Circle areas are proportional to degrees (i.e., numbers of host-plant species) of butterfly families. (**b**) The same plot for butterfly subfamilies. Circles represent butterfly subfamilies, with their colours corresponding to those given for butterfly families in (**a**). (**c**) Relationship between the compound contributions of plant species and degree (i.e., number of butterflies consuming the respective plant species). Circles represent plant species. Circle areas are proportional to the number of common specific compounds registered in the KNApSAcK database. Plant species in the Poaceae, Fabaceae, Brassicaceae and Rutaceae families are indicated by light green, dark green, yellow, and orange, respectively. (**d**) Relationship between the compound contributions (χ_*i*_) of different plant families and average neighbour degree (i.e., average number of host-plant families used by herbivorous butterflies on the respective plant species). Circle areas are proportional to the average number of common specific compounds. Colours are the same as in (**c**).

We focused on the statistics for each butterfly family for further evaluation. In particular, we used the average family-based evenness for butterfly family *i, E*(*i*), and the average number of compounds shared among host plants by butterfly species *k, N*_*s*_(*k*). Figure 5a shows the relationship between *Z*-scores of average plant family-based evenness for butterfly families, *Z*^*E*(*i*)^ = (*E*^real^(*i*) – *E*^rand^(*i*))/SD_*E*(*i*)_^rand^, and the *Z*-scores of the average number of common specific compounds in host plants, *Z*^*Ns*(*k*)^ = (*N*_*s*_^real^(*k*) – *N*_*s*_^rand^(*k*))/SD_*Ns*(*k*)_^rand^. *E*^real^(*i*) [*N*_*s*_^real^(*k*)] and *E*^rand^(*i*) [*N*_*s*_^rand^(*k*)] correspond to *E*(*i*) [*N*_*s*_(*k*)] obtained from the real-world network and randomized networks, respectively. SD_*E*(*i*)_^rand^, and SD_*Ns*(*k*)_^rand^ are the standard deviations for *E*^rand^(*i*) and *N*_*s*_^rand^(*k*), respectively (Data and Methods). Figure 5b is the same scatterplot for the butterfly subfamilies. For butterfly families and subfamilies, the *Z*-scores of evenness were mostly negative, which indicated that the actual host-plant relationship was more selective than random. The *Z*-scores of *N*_*s*_ were positive for the butterfly families Pieridae, Lycaenidae, and Papilionidae as well as for many subfamilies, which indicated that the host plants possessed more common specific compounds than would be expected by random chance. As clearly shown in Fig. 5a and b, the *Z*-scores of evenness and those of *N*_*s*_ were negatively correlated, indicating that the common specific compounds positively contributed to host-plant selection. For example, Pieridae and its subfamilies (Pierinae and Coliadinae) are significantly lower in evenness and higher in *N*_*s*_ compared with other families and subfamilies. Together with the previously known phenomenon that many phytophagous insects are highly adapted to defense chemicals produced by plants and use them as the specific host-finding cues^14^,^15^, the results obtained in this study provide quantitative support for our hypothesis that insects tend to selectively feed on closely-related plants sharing common specific chemical compounds.

We assumed that the global trend of host-plant selectivity was a mixture of different levels of feed-on-compound effects, i.e., some plants express a strong feed-on-compound effect, whereas others do not. Based on this idea, we determined the compound contribution of each plant species *i*, denoted as χ_*i*_ (see Methods). According to our calculations, most plant species had a positive value of χ_*i*_, thus contributing positively to the feed-on-compound effect (Fig. 5c and d).

The relationship between χ_*i*_ and degree (i.e., the number of butterflies that predate on the respective plant species) is shown in Fig. 5c. Two Rutaceae species (*Zanthoxylum ailanthoides* and *Phellodendron amurense*) exerted particularly high feed-on-compound effects, whilst a species in Poaceae (*Oryza sativa*) had a negative effect. We found that χ_*i*_ was moderately positively correlated with degree (Spearman’s rank correlation coefficient *r*_*s*_ = 0.53, *p* < 2.2 × 10^–16^). This correlation indicated that the presence of common specific plant compounds generally attracts phytophagous butterflies, an observation that supports the feed-on-compound hypothesis.

Figure 5d shows the relationship between plant family χ_*i*_ and average neighbour degree (i.e., the average number of host-plant families consumed by the butterfly herbivores of the respective plant species). Although the figure indicates that most plant families are eaten by mono-family-phagous butterflies (lower neighbour degree), we also observed that poly-family-phagous butterflies tend to feed on host plants with a lower χ_*i*_. This result reveals the relationship between χ_*i*_ and host selectivity.

### Common specific compounds in host plants

To identify specific compounds shared by host plants of a particular butterfly family, we used Fisher’s exact test, which, analogous to its application in genome-wide association studies, is the simplest method for inferring associations or correlations. We extracted all plant–herbivore relationships involving at least one plant compound registered in KNApSAcK^25^ from the original plant–herbivore network mentioned in the previous section, thereby obtaining 890 plant–herbivore relationships encompassing 217 butterfly species, 406 plant species and 3,507 plant compounds. Statistical significance based on Fisher’s exact test was evaluated using Cramer’s V; for example, given a sample size of 890 and a Cramer’s V value of ~0.6, the threshold *P*-value was set to 1.0 × 10^−6^.

**Figure 6:**
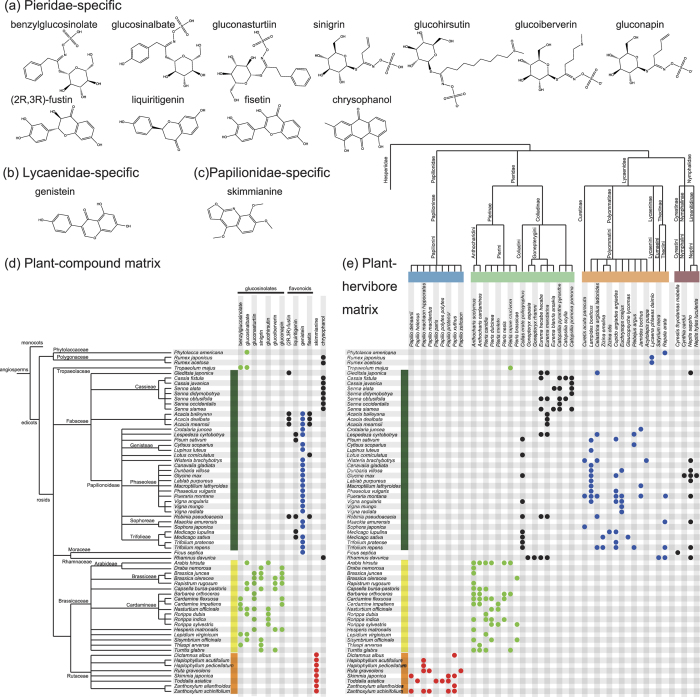
Compounds specific to host plants of a given butterfly family. (**a**–**c**) Compounds specific to host plants of Pieridae (**a**) Lycaenidae (**b**) and Papilionidae (**c**) families. (**d**) Matrix representation of plant species possessing the compounds shown in (**a**), (**b**) and (**c**). Among Pieridae-specific plant compounds, glucosinolates are represented by green dots; all others are represented by black dots. Lycaenidae-specific and Papilionidae-specific compounds are indicated by blue and red dots, respectively. (**e**) Plant–herbivore matrix of the plant species in (**d**) and the butterfly species that consume them.

**Figure 7:**
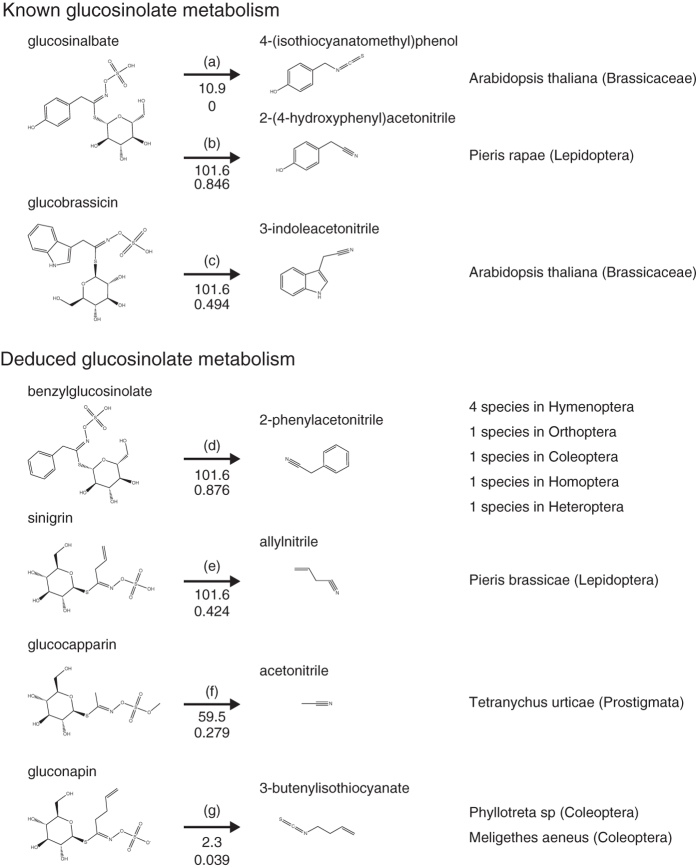
Known and deduced plant–herbivore metabolism of glucosinolates. Numbers below arrows indicate predictive values generated by E-zyme and E-zyme2 webservers. (**a**) *Arabidopsis thaliana* (plant family Brassicaceae) produces glucosinalbate. When plants are consumed by herbivores, the plant enzyme myrosinase acts on this compound to produce a toxic isothiocyanate compound. (**b**) *Pieris rapae*, a Pierinae butterfly, can prevent the production of the toxic isothiocyanate compound and instead produces a nitrile compound by altering the myrosinase activity with the help of nitrile-specifier protein (NSP). (**c**) *Arabidopsis thaliana* plants can also produce nitrile compounds from glucosinolates. (**d**–**f**) Putative chemical transformations of plant compounds (from KNApSAcK and Pherobase) to insect compounds (from Pherobase) identified by chemical structural comparison. The predicted transformations included a deduced reaction producing a compound possessed by a butterfly species (**e**).

Common specific compounds in host plants of Pierinae (family Pieridae), Lycaenidae, and Papilionae (family Papilionidae) butterflies are shown in Fig. 6a,b and c, respectively. Host plants of the Pierinae specifically possess a number of glucosinolates and flavonoids (Fig. 6a), whereas Papilionae (family Papilionidae) and Lycaenidae host plants are characterised by skimmianine and genistein, respectively (Fig. 6b,c). Among these identified compounds, glucosinolates are attractive to certain members of Pierinae but repel other butterflies^10^,^11^,^26^. Some plants, such as members of Brassicaceae, have a glucosinolate-myrosinase chemical defence system (Fig. 7a); to avoid this system, some Pieridae butterflies have a nitrile-specifier protein (NSP) (Fig. 7b)^14^. In addition, skimmianine (Fig. 6c) has been reported to attract certain Papilionidae butterflies, whereas a study on the interactions between Papilionidae and Rutaceae families^27^ has revealed that skimmianine has a prohibitive effect on these other butterflies. As clearly shown in the plant-compound matrix in Fig. 6d, plants belonging to the Brassicaceae and Rutaceae families typically possess glucosinolates and skimmianine, respectively. We found that genistein is specific to host plants of Lycaenidae butterflies; however, those plants belong to the family Fabaceae, which hosts a wide variety of Lycaenidae, Nymphalidae and Pieridae butterflies (Fig. 6e). Taking all of these results into consideration, we concluded that the identified common specific compounds contribute to the host-plant selectivity of mono-family-phagous butterflies.

### Enzyme catalysis in plant–herbivore relationships

Because herbivorous butterflies incorporate plant chemicals whilst feeding, an herbivorous butterfly and its host plants can be generally assumed to share more compounds (C1 in Fig. 1g) than other butterfly–plant pairs. This assumption is consistent with the results of previous studies. For example, some insects have been found to use plant-derived chemicals as signalling materials or as precursors of chemical messengers (e.g., pheromones)^15^. In another study, volatile organic compounds produced by insects and plants had an overlap of 87%. Lepidoptera (butterflies and moths) members share significantly more aromatics than do Hymenoptera (bees and ants), whilst no significant difference in shared monoterpenes has been observed between the two orders^28^.

In this study, we expanded the above assumption to compounds not shared between butterflies and their host plants (C2, C2′, C5 and C6 in Fig. 1g). Even when compounds in a butterfly are not exactly the same as those in host plants, they may be derived from the latter (C2 and C2′ in Fig. 1g) via the insect’s metabolism. To overcome this problem, we used the Pherobase^29^ and KNApSAcK^25^ databases to find potential substrate–product pairs for all possible combinations of compound pairs (see Methods for details)^30^,^31^.

Our database search revealed four insect compounds that could possibly be such products (right-hand side of Fig. 7d–g). The corresponding substrates of all four compounds (Fig. 7d–g, left) are present in Brassicaceae plants; of these four compounds, three (Fig. 7d,e and g, left) were found to be Pieridae host-plant specific (Fig. 6a). We used the E-zyme webserver (see Methods) to search for known enzymatic reactions having the same chemical transformation patterns as the query reaction^32^,^33^. Only one of the four reactions (Fig. 7g) was shown to have the same chemical transformation pattern as the known glucosinolate-myrosinase reaction (Fig. 7a). The remaining three reactions (Fig. 7d–f) had the same chemical transformation patterns as those of an avoidance reaction (Fig. 7b,c)^11^,^26^. Among the possible products (Fig. 7d–g, right), only allylnitrile (Fig. 7e, right) was found to be possessed by a butterfly species, *Pieris brassicae*. No butterfly species possessed any other products, according to Pherobase. Although there is no direct evidence, we showed that the integration of plant–insect relationships, plant compounds and insect compounds provides indirect evidences to infer potential enzyme catalysis. If we integrate additional data, i.e., gene or protein sequence information, further analysis would be available.

To cope with glucosinolates, Pieridae family butterflies have developed the NSP protein^34^. In the presence of NSP, insect myrosinase acts on glucosinolates to produce nitrile compounds (Fig. 7b,c) that are less toxic than the isothiocyanate compounds produced when NSP is absent (Fig. 7a). These two alternative reactions correspond to the reactions predicted in Fig. 7 (i.e., the reactions in Fig. 7d–f and Fig. 7g correspond to the myrosinase reaction in the presence or absence of NSP, respectively). This NSP protein is not found in any other organisms and is thought to be specific to Pieridae family butterflies.

We therefore searched genome and transcriptome sequences to determine whether myrosinase and NSP genes are present in a wider range of insects. At the time of our survey, the National Center for Biotechnology Information (NCBI) database contained 17 complete and 36 draft genome sequences of insect species, whilst Sequence Read Archive (SRA)^35^ had genome and transcriptome sequences for 48 and 131 insect species, respectively. Although some protein sequences of Pieridae butterflies have been registered at NCBI, none of their genomes have yet been sequenced. We conducted a comprehensive search for myrosinase and NSP against all published insect genomic and transcriptomic data (see Methods). Myrosinase-like sequences were detected in all sequenced butterflies and moths (*Bombyx mori, Chilo suppressalis, Danaus plexippus, Graphium sarpedon, Heliconius melpomene, Manduca sexta, Melitaea cinxia, Papilio glaucus, Papilio machaon, Papilio memnon, Papilio polytes, Papilio xuthus* and *Plutella xylostella*), but no NSP-like sequences were detected. This result is consistent with butterfly host selection of glucosinolate-containing plants^11^.

## Discussion

We have demonstrated that insects selectively feed on closely-related plants containing common specific compounds. Nevertheless, distinguishing between taxonomic and compound effects on plant–herbivore interactions (or host-plant selection) is difficult because plants in the same family may share many compounds. To overcome this complication, more careful examinations may be required. In particular, phylogenetic signals must be removed when evaluating the association between chemical compounds and plant–herbivore interactions. In this context, though comparative phylogenetic analysis^36^ is useful, this approach generally considers a simple evolutionary model in which species traits change in a Brownian fashion on a phylogenetic tree with accurate branch lengths. Phylogenetic comparative analysis was not applied in this study for two reasons. First, the phylogenetic tree was not accurate; thus, a phylogenetic comparative analysis may have led to misleading conclusions. Second, our dataset included only a few samples of specific plant–herbivore interactions and plant–compound relationships. In comparative phylogenetic analysis, a loss of statistical power is known to occur in small datasets^37^. Although definite conclusions were not obtained, we expect that the effect of compounds on plant–herbivore interactions is dominant^14^,^15^. In particular, only weak (although significant) phylogenetic signals have been observed in studies of plant–pollinator^38^ and seed-dispersal^39^ networks.

Our study has shown that the common specific compounds are mostly toxic compounds (Fig. 6), however, such common specific compounds were not found in Poaceae or Fabaceae, either of which is shared by some butterfly subfamilies in different families (Figs 3 and 4). This result may be due to the limited information in the examined databases but may also be explained by another hypothesis: Poaceae and Fabaceae do not possess particularly toxic compounds, and these less toxic plant species may serve as stepping stones during a transition to new host plants, because the butterflies were able to use their existing detoxification mechanisms to adapt to these plants. At least in Papilionidae, the diversification of butterfly species is thought to be correlated with changes in host plants^9^. *Papilio machaon*, one of many Papilionidae butterfly species that strongly depend on Rutaceae plants (Fig. 4), is thought to have evolved recently and feeds on a wide range of host plants, including Apiaceae. Rather than expanding to Apiaceae from Rutaceae plants in a single bound, a reasonable hypothesis is that *Papilio machaon* temporarily adopted less toxic hosts, such as Fabaceae plants, during its evolution.

When investigating host plant selection in this study, we only considered plant compounds; however, many other factors (such as hardness, leaf shape, and especially trichomes) are known to affect host plant edibility. Although these factors are described in field guides, encyclopaedias, and databases, their comprehensive and quantitative acquisition for use in informatics analysis is difficult. Moreover, the effects of phylogenetic signals were not considered in this study despite the importance of phylogenetic comparative analysis^40^. This is because an accurate phylogenetic tree is still unavailable. However, the absence of this analysis poses little problem because several studies have reported that phylogenetic signals are weak in ecological networks^41^,^42^. Despite these limitations, we have successfully shown that plant compounds generally contribute to the host-plant selection of phytophagous butterflies. Although molecular phylogeny is a powerful tool for understanding evolution, we strongly maintain that the integration of various types of data currently scattered across published articles and databases would enhance the study of natural evolutionary diversity.

### Concluding remarks

Diversity is one of the most appealing characteristics of insects. Because of this diversity, however, unstructured knowledge is scattered throughout the literature and various databases, hindering comprehensive and systematic analyses. The strength of the current study is the integration of various types of data, including those pertaining to plant–herbivore relationships and plant–compound relationships, which enabled us to obtain a list of compounds that putatively contributed to host plant selection. By itself, this approach is insufficient to enable conclusions, but it can provide insights about molecular mechanisms drawn from the relationship data. Further enrichment of these relationship data would enhance and widen the scope of possible analyses, such as the prediction of host plant relationships and the inference of responsible genes or proteins using various machine-learning techniques.

## Data and Methods

### Plant–herbivore relationships

Although the HOSTS database (http://www.nhm.ac.uk/research-curation/research/projects/hostplants/) maintained by the Natural History Museum in the UK contains information on plant–herbivore relationships involving butterflies and their host plants worldwide, it lacks data on some relationships, especially those in the Japanese Islands. We therefore conducted a literature survey to collect plant–herbivore relationship information relevant to the Japanese Islands. The data collected and used in this study are summarised in the InsectInDB database (http://insect-plant.org/). The current InsectInDB release (as of January 2016) contains approximately 545 insects, 1,922 edible plants and 3,435 plant–herbivore relationships. InsectInDB provides links to plant–herbivore relationships and metabolomic and genomic data via the scientific names of insects or plants.

### Plant-compound and insect-compound relationships

We collected plant compound data (Fig. 1c) using the KNApSAcK database^25^. We used Pherobase (http://www.pherobase.com), a major online resource cataloguing pheromone compounds that influence the behaviour of insects and other animals^29^, for the analysis of compounds common to insects and their host plants (Fig. 1g).

### Interaction frequency between insect and plant families

To evaluate the strength of the interaction between insect family *i* and plant family *j*, we computed *W*_*ij*_, the number of interactions between the insects in family *i* and the plants in family *j* appearing in the plant–herbivore network. We also evaluated the statistical significance of *W*_*ij*_ in the observed network using values from randomised networks (see below for details).

### Randomised networks and significance

A comparison with randomised model networks was performed to evaluate the statistical significance of the observed network measures^24^,^43^,^44^,^45^. We used the null model 2 46, which is similar to the fixed row-fixed column null model used by Bascompte *et al*.^47^. This null model generates random bipartite networks with degree sequences identical to those of real networks. In the null model, the probability that a plant connects to an animal is proportional to the product of node degrees for a given plant or animal.

The significance of the network measure *X* was evaluated based on the *Z*-score: *Z*_X_ = (*X*_real_ – *X*_rand_)/SD_rand_, where *X*_real_ is the network measure of a real-world network, *X*_rand_ is the average value of the network measure, and SD_rand_ is the standard deviation obtained from 1,000 randomised model networks.

### Family-based evenness index

To measure the diversity of host plants of a specific insect category, we used a family-based evenness index based on Pielou’s evenness index^48^, which is a Shannon diversity index used to evaluate species evenness. In particular, the evenness index of insect category (species, subfamily or family) *i* was defined as follows:


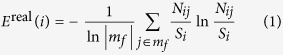


where *m*_*f*_ indicates the set of plant families appearing in the plant–herbivore network and |*m*_*f*_| is the number of plant families. *S*_*i*_ and *N*_*ij*_ are the number of plants interacting with insect category *i* (i.e., node degree of insect category *i*) and the number of plants in family *j* connecting to insect category *i*, respectively.

To characterise the overall trend of the evenness, we also considered the average family-based evenness: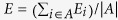, where *A* and |*A*| denote the set and number of insect species, respectively.

### Compound contribution to plant–herbivore networks

Drawing on terminology established for flavour networks^24^, we defined a coefficient of compound contribution, χ_*i*_, to evaluate the contribution of plant *i* to the feed-on-compound effect in plant–herbivore networks as follows:


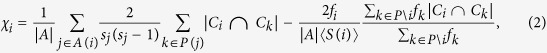


where *A* and *P* denote sets of insect and plant species, respectively, *A*(*i*) and *P*(*j*) are the set of insects feeding on plant *i* and the set of plant species consumed by insect *j*, respectively, |*X*| is the size (number of elements) of set *X, C*_*i*_ is the number of compounds in plant *i, f*_*i*_ represents the number of insects feeding on plant *i*, and *S (i*) is the average number of plant species that serve as prey in *A*(*i*). The first term indicates the average degree of the overlap of compounds between plant *i* and other plants in *A*(*i*) for the observed data. The second term is the expected value of the average degree obtained when assuming no host plant selectivity (i.e., insects feed on randomly selected plants). Thus, a positive or negative χ_*i*_ indicates the positive or negative contribution, respectively, of plant *i* to the feed-on-compound effect.

### Chemical compound-based reaction prediction

The reaction prediction based on chemical compounds consists of two steps. The first step is the problem of enzymatic reaction-likeness, i.e., whether a pair of metabolic compounds can be the substrate and product of a single enzymatic reaction^30^. The second step is the prediction of the actual enzymes responsible for the putative enzymatic reactions^33^. To accomplish the first step, we used known chemical transformation patterns encoded as SMIRKS strings^49^ to search for pairs of metabolic compounds potentially possessing the same chemical transformation pattern. For the second step, we used the E-zyme^32^ and E-zyme2^33^ webservers. E-zyme predicts putative enzyme classifications (referred to as Enzyme Commission numbers) with generated values reflecting the similarity between the given transformation patterns and known patterns. E-zyme2 predicts putative enzyme orthologues (i.e., homologous proteins/genes thought to have the same function). The values output by this program reflect the similarities of the transformation patterns as well as those of the surrounding conserved substructures.

### Massive-scale sequencing data

DNA and RNA sequences were obtained from NCBI (http://www.ncbi.nlm.nih.gov/) and DDBJ SRA (http://trace.ddbj.nig.ac.jp/dra/index_e.html) databases^35^. Raw RNA-sequencing data for each insect were downloaded from SRA and then assembled with Trinity^50^, one of the most effective RNA-sequencing assemblers. The obtained Trinity contigs were used as training datasets in the AUGUSTUS program^51^ to predict protein-coding sequences in the corresponding insect genome downloaded from NCBI. This analysis generated a list of putative protein sequences encoded in the corresponding insect genomes.

### Data Availability

The data collected in this study are summarised in a publicly accessible database, InsectInDB (http://insect-plant.org).

## Additional Information

**Publisher's note:** Springer Nature remains neutral with regard to jurisdictional claims in published maps and institutional affiliations.
